# Exploratory transcriptomic analysis suggests candidate genes associated with loss of response to ustekinumab in Crohn’s disease

**DOI:** 10.3389/fgene.2026.1812181

**Published:** 2026-05-22

**Authors:** Jiayi Lin, Tingting Xie, Jiahao Zhong, Yanmei Tu, Chang Xiao, Xueying Huang, Yixi Wang

**Affiliations:** The Second Affiliated Hospital, School of Pharmaceutical Sciences, Guangzhou Medical University, Guangzhou, China

**Keywords:** Crohn’s disease, loss of response, RNA-seq, transcriptomic, ustekinumab

## Abstract

**Background:**

Despite the favorable safety and efficacy of ustekinumab in clinical practice for Crohn’s disease, a subset of Crohn’s disease patients still experience loss of response to this treatment. This study was thus conducted to investigate the differential gene expression underlying ustekinumab loss of response in Crohn’s disease patients.

**Methods:**

This prospective study, grouped by response to ustekinumab, collected peripheral blood mononuclear cells from refractory moderate-to-severe adult Crohn’s disease patients (8 vs. 9) who were admitted to the Second Affiliated Hospital of Guangzhou Medical University and received initial ustekinumab treatment between January 2024 and June 2025 for RNA sequencing. For exploratory pathway analysis, differentially expressed genes identified by DESeq2 using exploratory thresholds (|FC| > 1.5, *P* < 0.05) were subjected to GO and KEGG enrichment, WGCNA, and PPI network analyses. Additionally, another 16 patients were included for the preliminary validation of candidate genes by qPCR. Furthermore, we conducted a clinical influence factor analysis on 81 patients who had already received medication.

**Results:**

RNA sequencing identified 510 differentially expressed genes between ustekinumab responders and non-responders. Trait association analysis showed that the cyan module was the module most strongly associated with both ustekinumab non-response and stricturing behavior, consistent with the independent influence of stricturing behavior identified in the retrospective clinical study. This module was enriched in platelet activation, neutrophil extracellular trap formation, and focal adhesion (KEGG), as well as coagulation and platelet aggregation (GO). Among the 10 candidate genes selected for validation, FFAR2, ITGA2B, SOCS3, and KCNJ15 exhibited statistically significant differential expression.

**Conclusion:**

Our findings suggest that loss of response may be potentially associated with pro-inflammatory pathways independent of the drug’s action pathways, as well as a “pro-fibrotic immune microenvironment” linked to intestinal strictures. Additionally, preliminary validation indicated that FFAR2, ITGA2B, SOCS3, and KCNJ15 may provide new perspectives for candidate differentially expressed genes associated with ustekinumab loss of response.

## 1 Introduction

Inflammatory bowel disease (IBD) is a chronic inflammatory disorder driven by dysregulated immune responses in genetically susceptible individuals, encompassing Crohn’s disease (CD) and ulcerative colitis (UC) (Ng et al., 2016). Intestinal stricture or penetrating disease develops in at least 50% of patients during the course of CD. As a heterogeneous, multifactorial disorder, CD is primarily characterized by dysregulated immune responses to diverse microbial antigens (Cohen et al., 2016; Feagan et al., 2016; Surace and Hedrich, 2019). This condition not only diminishes the quality of life for affected individuals but also incurs significant medical expenses and demands extensive healthcare resources. Consequently, CD has emerged as a topic of considerable public concern (Plaza et al., 2024). Biologic therapies, including ustekinumab (UST), have become cornerstone treatments for moderate to severe IBD. UST, a humanized monoclonal antibody targeting the p40 subunit of IL-12/23, blocks Th1- and Th17-mediated inflammatory pathways and has demonstrated significant efficacy in CD (Chaparro et al., 2022). However, a substantial proportion of patients experience loss of response (LOR) over time (Barré et al., 2018). The mechanisms underlying LOR are complex, involving drug immunogenicity, pharmacokinetic variability, and disease heterogeneity (Kwon et al., 2025; Wang et al., 2025a). Up to 30% of patients experience UST LOR, highlighting the need to identify predictive biomarkers (Feagan et al., 2016; Wu et al., 2022; Meserve et al., 2022; Xu et al., 2024; Yan et al., 2022). Due to heterogeneity in study populations and methodologies, findings on risk factors for UST LOR remain controversial. Moreover, current evidence is insufficient to fully define the real-world effectiveness of UST in CD, and data on molecular determinants of treatment responsiveness remain scarce. Previous transcriptomic studies have attempted to address this issue yet remain limited in scope. Few have applied bioinformatic analyses to characterize UST LOR in patients with CD. Moreover, most existing transcriptomic studies on UST LOR in IBD have relied on publicly available GEO datasets, which nevertheless provide valuable and reliable experimental data. For example, Manrong He et al. analyzed the GSE112366 dataset in their research, which had originally focused on the role of microvilli structural genes when considering treatment response (He et al., 2021; VanDussen et al., 2018). While these studies provide valuable insights, transcriptomic explorations of UST LOR remain extremely limited, and performing sequencing-based validation and exploration in real-world patient cohorts warrant further investigation. Transcriptomic sequencing using new cohorts from diverse ethnic populations can provide additional insights into this field. Furthermore, transcriptomic studies related to UST treatment response have often analyzed CD and UC collectively, without stratifying by disease subtype. The findings from these studies generally lack independent experimental validation, and validation at the cellular and animal experimental levels is even rarer. Another limitation of existing studies lies in the sample types used. In previous studies, predictive gene signatures for biologics were predominantly identified using intestinal epithelial tissue samples. However, the collection of these samples through colonoscopy and biopsy can cause significant discomfort to patients. Furthermore, peripheral blood mononuclear cells (PBMCs) offer a minimally invasive approach to capture systemic immune signatures, yet they remain underexplored in the context of UST LOR in patients with CD. Compared with intestinal tissue samples, PBMCs also present greater feasibility and convenience as biomarkers, highlighting the value of further investigation using this sample type.

In summary, a key challenge in the management of IBD is identifying patients who will respond to biologic therapy. However, research on predictive factors for UST LOR in patients with CD remains relatively limited. In recent years, driven by declining costs of RNA sequencing (RNA-seq), improved analytical pipelines, and advanced bioinformatic tools, transcriptomics has become increasingly powerful in studying immune-mediated diseases, with treatment response prediction playing a critical role in therapy selection. In this preliminary exploratory study, we performed RNA-seq transcriptomic analysis on pre-treatment PBMCs from CD patients undergoing UST therapy. Combined with bioinformatics analyses including GO, KEGG, WGCNA and PPI networks as well as clinical data, we aimed to identify differentially expressed genes (DEGs) and signaling pathways associated with UST LOR, explore its potential mechanisms, and carry out preliminary experimental validation. This exploratory study may provide a novel perspective for future research on the predictive factors and underlying mechanisms of UST LOR in patients with CD.

## 2 Materials and methods

### 2.1 Patients and study selection

This prospective study recruited two independent cohorts of adult patients with refractory moderate-to-severe CD who received UST treatment for the first time at the Department of Gastroenterology, Second Affiliated Hospital of Guangzhou Medical University, between January 2024 and June 2025. For RNA-seq, PBMCs were collected from 9 UST responders and 9 UST non-responders. For qPCR validation, PBMCs were independently collected from another 8 UST responders and 8 UST non-responders, with no overlap between the two cohorts. Baseline blood sampling for PBMC transcriptomic processing was performed prior to UST initiation in both cohorts separately. In addition, we conducted a clinical influencing factor analysis on 47 responders and 34 non-responders among patients who had previously received medication.

Due to the clinical rarity of the non-responder phenotype meeting our strict monotherapy criteria, all nine eligible non-responders with high-quality RNA samples were included. To minimize baseline confounding factors, nine definitive responders were purposively selected from the eligible pool to match the non-responder group at the cohort level in terms of baseline demographics and inflammatory status. The comparability of the resulting cohorts is detailed in Supplementary Table S1. The same selection strategy was applied to the qPCR validation cohort, and the comparability of these two groups is detailed in Supplementary Table S2.

The inclusion criteria for transcriptomics were CD patients aged over 18 years with moderate-to-severe disease who received UST treatment for the first time and underwent regular follow-up. The exclusion criteria included patients with incomplete baseline data or who were lost to follow-up during the study; patients with incomplete blood sample collection; patients receiving non-CD indication treatments; patients allergic to UST components or with active tuberculosis; patients with severe infections; patients with prior intestinal resection.

### 2.2 UST treatment course

Regarding the UST treatment regimen, all patients received weight-based intravenous induction (260 mg for body weight ≤55 kg, 390 mg for 55–85 kg, and 520 mg for >85 kg), followed by 90 mg subcutaneous injections at week 8 and every 12 weeks thereafter.

### 2.3 Rating and grouping criteria

Clinical scores: HBI score, with a total score ≤4 defined as remission.

Endoscopic score: SES-CD score, with a total score decrease >50% from baseline defined as endoscopic response in CD, and a total score of 0–2 defined as remission.

All enrolled patients were divided into the responder group (R group) and non-responder group (NR group) based on treatment efficacy after UST therapy.

Patients meeting any of the following criteria were assigned to the NR group: no clinical response at both week 8 and week 16/20; need for increased UST dosage or shortened dosing intervals due to persistent disease activity within 6 months of treatment initiation; switching to other IBD medications due to inadequate UST efficacy within 6 months; concurrent use of other IBD-related medications; undergoing IBD-related surgery or discontinuing treatment due to adverse events.

Patients who maintained the same medication regimen with sustained effectiveness from treatment initiation through month 6 were assigned to the R group.

Assessment timepoints were scheduled at weeks 0 (baseline), 8, 20, 32, and 44, corresponding to each UST administration visit. The follow-up endpoint for this analysis was set at week 44.

### 2.4 RNA extraction and RNA-seq analysis

#### 2.4.1 PBMCs isolation

Prior to treatment, a 3 mL blood sample was collected from each patient into an EDTA anticoagulant tube. PBMCs were isolated using density gradient centrifugation. Whole blood was diluted 1:1 with 1×PBS supplemented with 2%–10% FBS, then gently overlaid onto 3 mL of lymphocyte separation medium in a 15 mL centrifuge tube at a 2:1 ratio. After centrifugation at 800 *g* for 20–30 min at room temperature with acceleration and deceleration set to 30% maximum, the plasma layer was removed and the PBMC buffy coat was transferred to a new tube. Cells were washed twice with 10 mL 1×PBS containing 2%–10% FBS by centrifugation at 250 *g* for 10 min at room temperature. Subsequently, cells were lysed in 1 mL TRIzol reagent, snap-frozen in liquid nitrogen, and stored at −80 °C to prevent repeated freeze-thaw cycles. Prior to detection, frozen samples were thawed at room temperature and sent to Gene Denovo Biotechnology Co., Ltd. for RNA-seq analysis.

#### 2.4.2 RNA extraction

Total RNA extraction from the TRIzol lysates, quality control, and subsequent library preparation were performed by Gene Denovo Biotechnology Co., Ltd. (Guangzhou, China). Total RNA was extracted from PBMCs using TRIzol reagent according to the manufacturer’s instructions. On-column DNase digestion was performed to remove genomic DNA. RNA concentration and purity were assessed using a NanoDrop 8000 spectrophotometer.

#### 2.4.3 Library construction and sequencing

The extracted mRNA was enriched using mRNA Capture Beads. After purification, the mRNA was fragmented at high temperature. The fragmented mRNA was then used as a template for first-strand cDNA synthesis in a reverse transcription enzyme mixture. During second-strand cDNA synthesis, end repair and A-tailing were performed. Next, adapters were ligated, and Hieff NGS® DNA Selection Beads were used for purification to select target fragments. PCR library amplification was then carried out, followed by sequencing on the Illumina Novaseq X Plus platform.

#### 2.4.4 RNA-seq data processing and quality control

Total RNA integrity was assessed using an Agilent 2100 Bioanalyzer, and only samples with an RNA integrity number (RIN) ≥ 7.0 were used for library preparation. Raw sequencing data were processed using fastp (version 0.18.0) for quality control. Reads with quality score <20 or length <50 bp were filtered out. After filtering, the proportion of low-quality reads was less than 1%, the Q30 scores of the filtered reads exceeded 90%, and the mapping rate to the reference genome was greater than 90%.

#### 2.4.5 Differential expression analysis

Clean reads were aligned to the human reference genome (GRCh38) using HISAT2 (v2.2.1), and gene-level read counts were quantified using featureCounts (v2.0.1). Differential expression analysis was performed using DESeq2 (v1.36.0). Genes with low expression (sum of counts <10 across all 18 samples) were filtered out. Given the exploratory nature of this study, we used a lenient threshold: genes with |log_2_FC| > 0.585 (FC > 1.5) and *P* < 0.05 were considered DEGs.

#### 2.4.6 Functional enrichment analysis

Functional enrichment analysis was performed using the online platform provided by Gene Denovo Biotechnology Co., Ltd. GO and KEGG enrichment analyses were conducted based on the identified DEGs, with Benjamini–Hochberg adjusted *P*-value <0.05 considered statistically significant.

#### 2.4.7 Co-expression network construction

Co-expression networks were constructed using the WGCNA (v1.47) package in R. After filtering genes, gene expression values were imported into WGCNA to construct co-expression modules using the automatic network construction function blockwiseModules with default settings, except that power = 8 (selected based on the scale-free topology criterion *R* ^2^ > 0.85), TOMType = “unsigned,” mergeCutHeight = 0.25, and minModuleSize = 50. Genes were clustered into 19 correlated modules.

#### 2.4.8 PPI network construction and hub gene identification

To explore the interactions of DEGs at the protein level, we constructed a PPI network. The overlapping DEGs between the responder and non-responder groups and the cyan module genes were selected and merged, resulting in 73 DEGs. The PPI network was constructed using the STRING database, restricting the organism to *Homo sapiens* and applying a minimum required confidence score of 0.4. Disconnected nodes were excluded from the analysis. The network was then imported into Cytoscape (version 3.6.1) for visualization and further analysis. Hub genes were identified using the cytoHubba plugin with the MCC (Maximal Clique Centrality) algorithm.

### 2.5 qPCR validation

**FIGURE 1:**
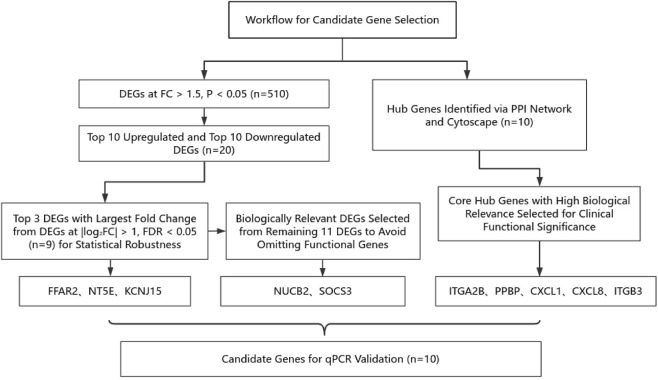
Workflow for candidate gene selection.

Based on a comprehensive selection strategy, the genes for qPCR validation were determined as follows: From the nine strict-threshold DEGs, three with the largest fold changes (NT5E, KCNJ15, FFAR2) were selected to ensure statistical robustness. From the remaining 11 candidate genes, two genes relevant to CD and UST (NUCB2, SOCS3) were selected to avoid missing potential signals. Meanwhile, using WGCNA combined with PPI network analysis, five hub genes located at core positions in the PPI network (ITGA2B, PPBP, CXCL1, CXCL8, ITGB3) were identified. Finally, a total of 10 genes were included for qPCR validation. The workflow is presented in Figure 1.

Based on the gene sequence information, qPCR amplification primers were designed using Primer 5 software. For the target gene mRNA sequences, the design parameters were set as follows: product length 50–150 bp, primer length 18–25 bp, and annealing temperature 60 °C ± 3 °C. The primer sequences are listed in Table 1.

**TABLE 1:** Oligonucleotide sequences used to quantify gene expression in qPCR.

Gene symbol	Forward (5′- 3′)	Reverse (5′-3′)
ITGA2B	GAT​CAG​TTT​GTG​CTG​CAG​TCG	AGC​TGT​GTC​CAC​ACC​TGA​GC
CXCL1	CTCTCACAGCCGCCAGAC	CACGGACGCTCCTGCTG
CXCL8	AGT​TTT​TGA​AGA​GGG​CTG​AGA	TGC​TTG​AAG​TTT​CAC​TGG​CA
ITGB3	ATT​GGA​GAC​ACG​GTG​AGC​TT	GAC​GAT​CAG​GCT​GTC​CTT​GA
NUCB2	GGC​TGG​AGG​ACA​GGT​TTG​TG	GAC​TCG​TAA​CAC​GTT​CTG​GC
SOCS3	GGAGGTGACGAGCCCCC	AAA​CTT​GCT​GTG​GGT​GAC​CA
KCNJ15	CAG​GCA​GTA​GCA​GAA​TCC​CA	TTT​CCC​CCA​CAT​TCC​TGG​TC
FFAR2	GGG​GCT​AAA​GCT​CTG​TTC​CT	CCG​GCA​GCA​TCC​TTG​TGT​T
NT5E	ACC​TGA​TTT​GTG​ATG​CAA​TGA​TTA	TGG​ATT​CCA​TTG​TTG​CGT​TCA
PPBP	TGG​CGA​AAG​GCA​AAG​AGG​AAA	CGA​CTT​GGT​TGC​AAT​GGG​TT
GAPDH	CTC​CGG​GTG​ATG​CTT​TTC​CT	GCC​CAA​TAC​GAC​CAA​ATC​AGA​G

Total RNA was extracted using TRIzol, quantified with a NanoDrop 8000, and reverse-transcribed to cDNA. qPCR was performed on a QuantStudio™ 5 system. The 20 µL reaction mixture contained 10 µL SYBR Green Master Mix, 0.4 µM each primer, and 2 µL cDNA template. The cycling conditions were: 95 °C for 30 s, followed by 40 cycles of 95 °C for 5 s and 60 °C for 30 s. Melting curve analysis was performed to verify specificity. Each sample was run in triplicate. Gene expression was analyzed using the 2^−ΔΔCT^ method. GAPDH was used as the internal reference gene. Differences between responders and non-responders were analyzed using unpaired two-tailed Student’s t-test after confirming homogeneity of variances by F-test. *P* < 0.05 was considered statistically significant.

### 2.6 Statistical analysis

Statistical analysis was performed using SPSS 22.0 and R. Normally distributed measurement data were expressed as $\bar{x}$ ± s, with intergroup comparisons conducted using independent samples t-test; non-normally distributed measurement data were expressed as M (Q1, Q3), with intergroup comparisons performed using nonparametric tests (Mann-Whitney U test); intergroup comparisons of categorical variables were conducted using χ^2^ test or Fisher’s exact test; enumeration data were expressed as cases (%).

### 2.7 Ethical considerations

All primary materials and peripheral blood samples were collected following the acquisition of written informed consent from all participants, and in strict accordance with the ethical principles of the World Medical Association Declaration of Helsinki. This study involving human patient samples was approved by the Ethics Advisory Committee of the Second Affiliated Hospital of Guangzhou Medical University (Approval No.: 2024-YJS-ks-27).

## 3 Results

### 3.1 Clinical independent influencing factors

Among the 81 retrospectively enrolled CD patients with regular follow-up, 34 patients experienced UST LOR, with an incidence rate of 41.98%. The detailed results of the analysis on influencing factors for UST LOR in patients with CD patients are presented in Table 2. Univariate analysis revealed that patients in the NR group had significantly higher levels of intestinal fistula (*P* = 0.005), intestinal stricture (*P* < 0.001), hs-CRP (*P* = 0.026), SAA (*P* = 0.008), ESR (*P* = 0.021), HBI score (*P* = 0.001), and SES-CD score (*P* < 0.001). In addition, the proportions of patients with baseline HBI score ≥7 (*P* = 0.001) and baseline SES-CD score >9 (*P* < 0.001) were significantly higher in the NR group than in the R group.

**TABLE 2:** General clinical data and comparison of CD patients treated with UST (n = 81).

Clinical characteristics	All (n = 81)	R (n = 47)	NR (n = 34)	*P* (R vs. NR)
Age [years, M (Q1, Q3)]	33.00 (23.50, 47.00)	33.00 (23.00, 41.00)	36.00 (24.75, 51.25)	0.163
Gender [cases, male/female]	49/32	27/20	22/12	0.510
Lesion location [cases (%)]	—	—	—	0.670
Terminal ileum (L1)	29 (35.8)	19 (40.4)	10 (29.4)	—
Colonic type (L2)	4 (4.9)	2 (4.3)	2 (5.9)	—
Ileocolonic type (L3)	45 (55.6)	25 (53.2)	20 (58.8)	—
Ileocolonic type with upper gastrointestinal tract involvement (L3 + L4)	3 (3.7)	1 (2.1)	2 (5.9)	—
Intestinal fistula [cases (%)]	13 (16.0)	3 (6.4)	10 (29.4)	0.005*
Perianal lesions [cases (%)]	20 (24.7)	8 (17.0)	12 (35.3)	0.060
Intestinal stenosis [cases (%)]	31 (38.3)	10 (21.3)	21 (61.8)	<0.001*
Baseline weight loss [cases (%)]	23 (28.4)	14 (29.8)	9 (26.5)	0.744
Previous biologic exposure [cases (%)]	27 (33.3)	12 (25.5)	15 (44.1)	0.080
Baseline hs-CRP [mg/L, M (Q1, Q3)]	4.90 (1.30, 40.40)	2.80 (0.80, 20.3)	15.80 (2.18, 67.40)	0.026*
Baseline SAA [mg/L, M (Q1, Q3)]	8.20 (5.00, 71.58)	5.48 (5.00, 42.26)	30.48 (5.55, 144.96)	0.008*
Baseline ESR [mm/h, M (Q1, Q3)]	21.00 (7.00, 49.50)	14.00 (6.00, 33.00)	31.50 (12.50, 55.75)	0.021*
Baseline HBI score [points, M (Q1, Q3)]	6.00 (5.00, 8.00)	6.00 (4.00, 8.00)	7.00 (6.00, 10.00)	0.001*
Baseline HBI score ≥7 points [cases (%)]	39 (48.1)	15 (31.9)	24 (70.6)	0.001*
Baseline SES-CD score [points, M (Q1, Q3)]	8.00 (6.00, 13.00)	7.00 (5.00, 8.00)	12.00 (7.75, 16.25)	<0.001*
Baseline SES-CD score >9 points [cases (%)]	33 (40.7)	10 (21.3)	23 (67.6)	<0.001*

R, responder group; NR, non-responder group; hs-CRP, high-sensitivity C-reactive protein; SAA, serum amyloid A; ESR, erythrocyte sedimentation rate; —, no available data in this category.

* p value < 0.05.

According to the statistical principle of at least 10 events per variable in multivariate logistic regression, among variables associated with UST LOR in univariate analysis, three clinically meaningful variables without multicollinearity were finally included: baseline HBI score ≥7, baseline SES-CD score >9, and intestinal stricture. Multivariate logistic regression showed that intestinal stricture was an independent risk factor for UST LOR (OR = 4.417, 95% CI: 1.436–13.592, *P* = 0.010), as shown in Table 3.

**TABLE 3:** Analysis of influencing factors for UST LOR in patients with CD (n = 81).

Factor	B	SE	Wald	*P*-value	OR (95% CI)
Baseline HBI score ≥7 points	1.132	0.602	3.538	0.060	3.101 (0.954–10.084)
Baseline SES-CD score >9 pionts	1.173	0.606	3.746	0.053	3.231 (0.985–10.593)
Intestinal stenosis type	1.486	0.573	6.71	0.01	4.417 (1.436–13.592)

B, regression coefficient; SE, standard error; Wald, Wald statistic; *P*-value, probability value.

Throughout the follow-up period of this retrospective study, the incidence of UST-related adverse events was only 4.94% (4/81). One patient developed a rash after the first dose, which resolved after anti-allergic treatment. One patient presented with dermatitis and cold-like symptoms, and another patient experienced dizziness; both cases resolved spontaneously. The above adverse events were all within a manageable category, with no severe adverse events observed.

### 3.2 Sample characteristics and sequencing data quality

A total of 34 patients with CD were prospectively enrolled in this study, including 18 patients in the RNA-seq cohort (9 vs. 9) and 16 patients in the qPCR validation cohort (8 vs. 8). In both cohorts, there were no significant differences in baseline characteristics between the responder and non-responder groups, including gender, age, stricture, baseline hs-CRP, SAA, ESR, WBC, Hb, ALB, baseline clinical scores, and endoscopic scores, indicating that the two groups were comparable. No severe adverse events directly attributable to UST were reported in these cohorts during the follow-up period. Details are shown in Supplementary Tables S1, S2.

### 3.3 Analysis of differences between R and NR group

#### 3.3.1 DEGs between R and NR group

**FIGURE 2:**
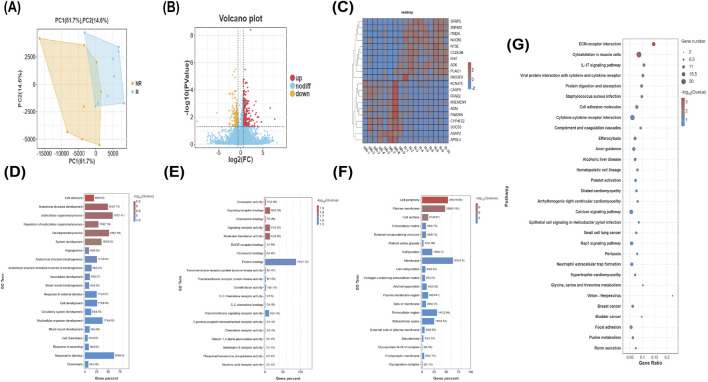
Comparison of DEGs between the R group and NR group. **(A)** PCA plot. **(B)** Volcano plot of DEGs. **(C)** Heatmap of the top 10 upregulated and top 10 downregulated DEGs. **(D–F)** Top 20 functional enrichments in BP, CC, and MF analyses. **(G)** Top 30 enriched KEGG pathways.

Principal component analysis (PCA) was conducted on the 18 samples included in the study. As illustrated in Figure 2A, the visualization indicated that one sample (PC1 = 25,429.08, PC2 = −6,644.21) significantly deviated from the other data points in the principal component space. To enhance the robustness of our analysis, we chose to exclude this outlier sample (NR-6) in subsequent analyses, thereby retaining 17 samples (R = 9, NR = 8) for further examination.

To determine an appropriate screening strategy for this exploratory study, we first applied the conventional stringent threshold (|log_2_FC| > 1 and FDR < 0.05), which yielded only nine DEGs—insufficient for subsequent pathway and network analyses. We therefore gradually relaxed the screening criteria and observed that the number of DEGs stabilized at approximately 510 under the conditions of FC > 1.5 (i.e., |log_2_FC| > 0.585) and *P* < 0.05. This approach is supported both by methodological literature (Liu et al., 2023; Warden and Wu, 2024) and by a highly similar study (Granot et al., 2023), which also used *P* < 0.05 and FC > 1.5 when no significant DEGs were found after FDR correction. To help assess reliability, we subsequently employed qPCR validation in an independent cohort and cross-analysis using WGCNA to intersect with DEGs. Adopting an overly stringent threshold might have risked omitting genes with subtle but biologically relevant expression changes.

We ultimately identified 510 candidate differentially expressed genes, including FFAR2, ITGA2B, SOCS3, and KCNJ15. Among these, 309 genes were overexpressed and 201 genes were underexpressed in non-responders, as illustrated in the volcano plot Figure 2B. A heatmap was generated for the top 10 upregulated and 10 downregulated genes, shown in Figure 2C.

#### 3.3.2 GO and KEGG pathway enrichment analyses

GO enrichment analysis of significant DEGs encompasses three independent domains: biological process (BP), cellular component (CC), and molecular function (MF). The specific results are illustrated in Figures 2D–F.

The top 30 enriched KEGG pathways are shown in Figure 2G, with ECM-receptor interaction, cytoskeleton in muscle cells, IL-17 signaling pathway, cell adhesion molecules, and focal adhesion being enriched in DEGs.

### 3.4 WGCNA analysis

**FIGURE 3:**
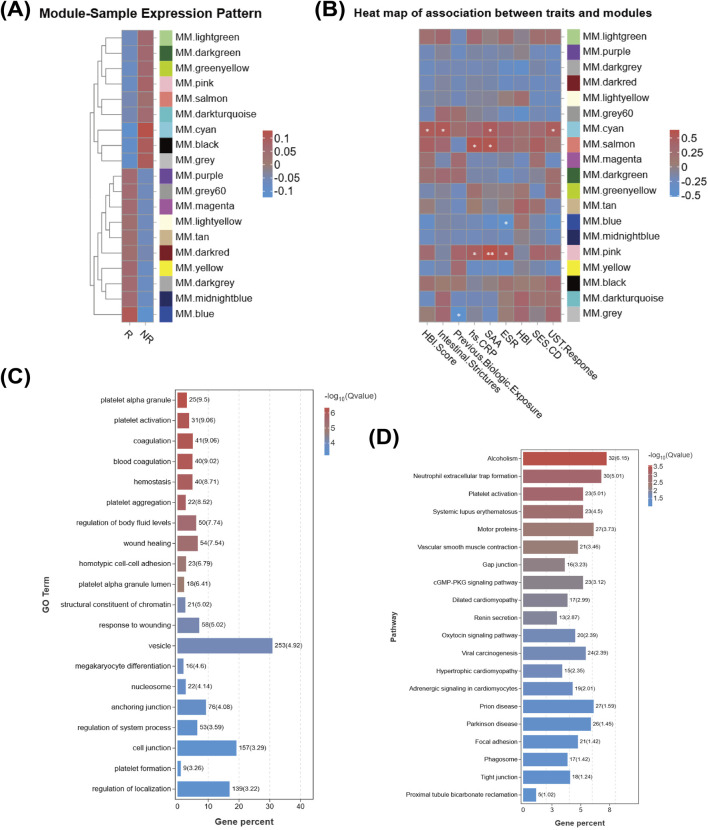
WGCNA, module-trait association, and functional enrichment analysis in UST LOR CD patients. **(A)** Module-sample expression pattern. **(B)** Trait-module association heatmap. **(C)** GO enrichment analysis of the cyan module (top 20 terms). **(D)** KEGG pathway enrichment analysis of the cyan module (top 20 pathways).

To further analyze the gene co-expression patterns associated with UST LOR in patients with CD, we employed WGCNA to model RNA-seq data from 17 patients (8 vs. 9). Figure 3A depicts the module-sample expression patterns across R and NR groups. Ultimately, in the module-gene relationship analysis, we found that the module most significantly associated with UST LOR was the cyan module (containing 922 genes) (correlation coefficient = −0.49, p-value = 0.046), as shown in Figure 3B. Notably, in the trait association analysis, we found the cyan module most strongly associated with UST LOR was also the module most correlated with stenosis, as illustrated in Figure 3B. This corresponds to the finding that stenosis is an independent influencing factor as identified in the first part. As shown in Figure 3C, GO analysis enriched terms related to platelet alpha granule, blood coagulation, platelet aggregation, and wound healing. KEGG analysis revealed significant enrichment of platelet activation, neutrophil extracellular trap (NET) formation, and focal adhesion, as displayed in Figure 3D. These results indicate that the cyan module is enriched in pathways linked to platelet activation, coagulation, and neutrophil-mediated inflammation, which are consistent with a pro-fibrotic and therapy-resistant microenvironment.

### 3.5 PPI network and hub genes

**FIGURE 4:**
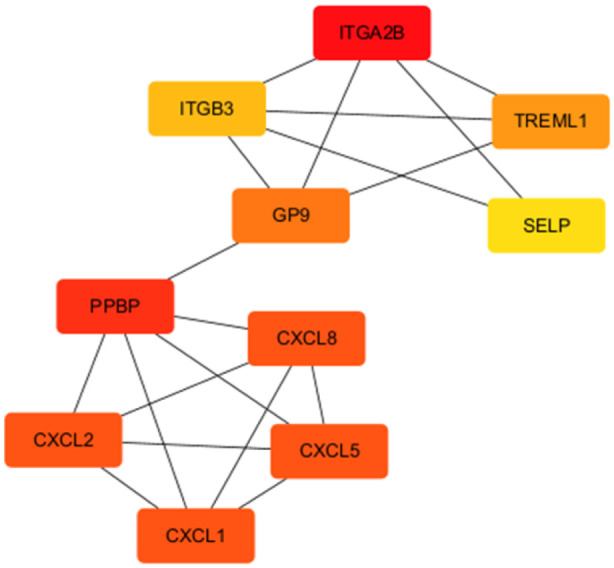
Hub gene PPI network.

As shown in Figure 4, the PPI network of the overlapping DEGs consisted of 72 nodes and 47 edges. Using the cytoHubba plugin, the top 10 genes with the highest degree values were identified as hub genes, including ITGA2B, CXCL2, TREML1, PPBP, CXCL1, CXCL8, GP9, SELP, ITGB3, and CXCL5.

### 3.6 Validation of candidate genes by qPCR

We performed preliminary validation in a cohort of 16 patients. As described above, we prioritized genes with clear biological significance and significant differences (e.g., log2FC > 1), and combined them with the hub genes obtained from the aforementioned PPI analysis, ultimately identifying 10 candidate genes (CXCL1, CXCL8, FFAR2, ITGA2B, ITGB3, NT5E, NUCB2, PPBP, SOCS3, KCNJ15).

**FIGURE 5:**
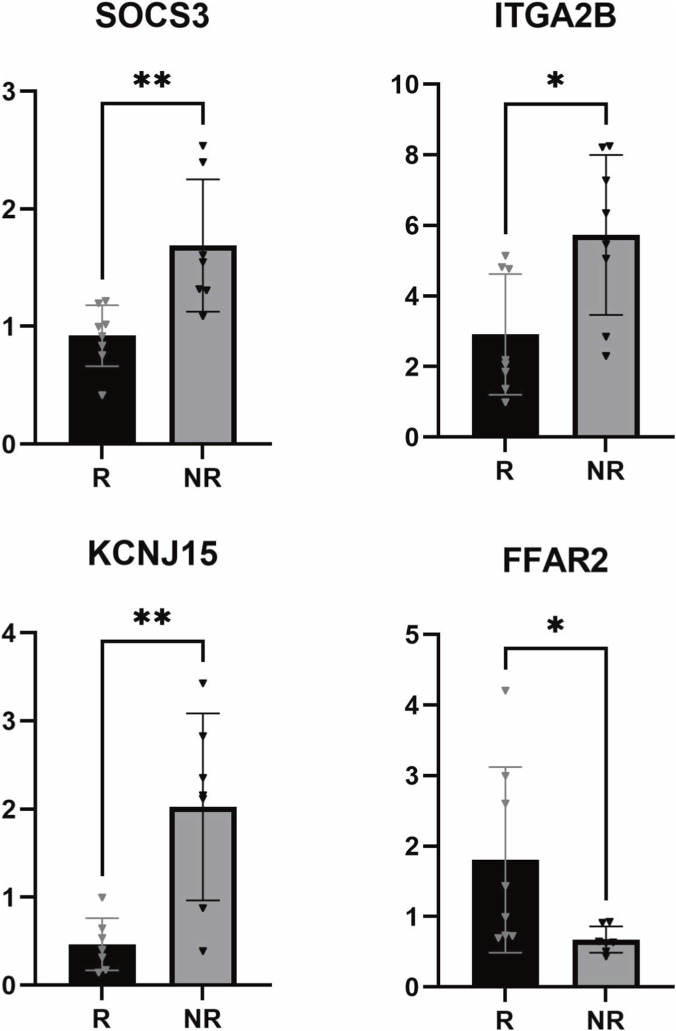
Preliminary qPCR validation. Note: R = responders, NR = non-responders; *p < 0.05, **p < 0.01.

The qPCR results showed that in the UST NR group at baseline, FFAR2 was significantly downregulated, while ITGA2B, SOCS3, and KCNJ15 were significantly upregulated with statistical significance. The expression differences of other genes did not reach statistical significance, but most expression trends were consistent with RNA-seq. ITGA2B, SOCS3, FFAR2 and KCNJ15 are shown in Figure 5, and the results for all other genes are provided in the Supplementary Figure S1.

## 4 Discussion

To investigate the factors influencing UST LOR in patients with CD, we first identified intestinal strictures as independent risk factors for UST LOR through a retrospective cohort clinical study. Furthermore, we constructed gene expression profiles for R group and NR group from the prospective cohort using RNA-seq and conducted transcriptomic analysis. Among the 510 differentially expressed genes ultimately identified, 15 were consistent with previously published literature (He et al., 2021; Nishioka et al., 2021; Pavlidis et al., 2022; Granot et al., 2023; Li et al., 2024; Xu et al., 2024). Among the top 10 up- and downregulated DEGs, three genes (CASP5, FAM20A, SOCS3) were consistent with previously reported DEGs in the literature. KEGG enrichment analysis revealed that the DEGs were primarily enriched in pathways including ECM-receptor interaction, cytoskeleton in muscle cells, IL-17 signaling pathway, cell adhesion molecules, and focal adhesion. These pathways are mainly associated with cell adhesion, extracellular matrix interactions, regulation of muscle cell cytoskeleton, and inflammatory signal transduction. In the trait-module association analysis, we identified the cyan module as the most relevant module for UST LOR and also the most correlated with stenosis, which aligns with the findings from our retrospective cohort study: stenosis is one of the independent influencing factors for UST LOR. Functional enrichment analysis revealed that the cyan module was enriched in pathways related to platelet activation, extracellular matrix interaction, and NET formation. Finally, we validated 10 candidate target genes via qPCR. The results showed that the expression trends of most genes were consistent with RNA-seq data, with FFAR2, ITGA2B, SOCS3, and KCNJ15 demonstrating statistical significance. To our knowledge, this is the first study to provide preliminary evidence that differential expression of FFAR2, ITGA2B, SOCS3, and KCNJ15 in PBMCs is associated with LOR to UST in CD patients.

SOCS3 serves as a critical intracellular negative feedback regulator of the JAK-STAT signaling pathway. The mechanism of action of UST involves the inhibition of the IL-12/IL-23 pathway, which relies on the JAK/STAT pathway for signal transduction. Our transcriptomic and qPCR analyses consistently demonstrated an upregulation of SOCS3 expression in patients classified as non-responders. Furthermore, among the ten hub genes identified through PPI network construction, CXCL8, CXCL2, CXCL1, CXCL5, and PPBP belong to the CXC chemokine family. Although CXCL8, CXCL1, and PPBP did not achieve statistical significance in our validation cohort, they displayed an upward trend that aligns with the RNA-seq results. These chemokines are primary downstream effector molecules directly induced by the IL-17 signaling pathway, which was also enriched in our KEGG analysis. Consistently, our GO enrichment analysis revealed significant enrichment of chemotaxis-related processes and chemokine-binding functions such as cell chemotaxis, chemokine binding, and interleukin-8 receptor activity. Moreover, the Cytokine-cytokine receptor interaction pathway, which includes CXCLs, IL-10, and SOCS3, was enriched in our KEGG analysis, supporting a broad chemokine-driven inflammatory network in UST non-responders. The cyan module showed enrichment of NET formation, which aligns with our observed elevation of CXC chemokines and chemotaxis-related GO terms. This suggests that neutrophil-driven inflammation is a key component of the chemokine network in UST non-responders. Li et al. analyzed the GSE207022 dataset (intestinal tissue samples) and identified SOCS3 as a key gene associated with UST response in CD patients, though no independent validation was performed. The study also revealed a significant increase in Th1 cells among non-responders, which is notable given the well-established role of Th1 cells in chemokine-driven inflammation (Li et al., 2024). Granot et al. reported that CXCL1, CXCL2, and CXCL3 were induced in PBMCs of UST non-responders (p < 0.05, FC > 1.5), with functional enrichment for CXCR chemokine receptor binding and IL-10 signaling (Granot et al., 2023). In another study (Pavlidis et al., 2022) on UC, it was found that the transcriptional module regulated by IL-22, including SOCS3, was enriched to a greater extent in UC patients who exhibited poorer responses to UST. This also suggests that SOCS3 may serve as a potential biomarker for the efficacy of UST therapy.

Based on our transcriptomic data and supporting evidence from the literature, we propose the following hypothesis regarding UST resistance in patients with Crohn’s disease. Our results demonstrate that SOCS3 is significantly upregulated in UST non-responders, together with multiple chemokines including CXCL1, CXCL2, CXCL3, CXCL5, and CXCL8. Functional enrichment analyses reveal that these chemokines are significantly enriched in GO terms related to chemotaxis, cellular chemotaxis, chemokine binding, and chemokine receptor activity. KEGG pathway analysis further identifies significant enrichment in the IL-17 signaling pathway and cytokine-cytokine receptor interaction. As SOCS3 is a potent negative regulator of the JAK/STAT pathway, we hypothesize that its excessive upregulation leads to oversuppression of JAK/STAT signaling. This not only attenuates pro-inflammatory signals but, more critically, also impairs anti-inflammatory cascades dependent on specific JAK/STAT subtypes, such as IL-10-mediated STAT3 activation. Since IL-10 normally suppresses the expression of multiple chemokines, compromised IL-10 signaling results in uncontrolled chemokine production. Consequently, persistent pro-inflammatory factors such as IL-22 and IL-17 may continue to drive chemokine release, leading to sustained neutrophil chemotaxis and accumulation of activated inflammatory cells. Under these conditions, the therapeutic effect of UST, which targets the p40 subunit of IL-12/IL-23, may be bypassed or overwhelmed, thus contributing to a poor clinical response despite continuous treatment.

Our study found that FFAR2 was significantly downregulated in non-responders, suggesting a possible loss of its anti-inflammatory function. FFARs have been proposed as targets for IBD, but only FFAR2 has been shown to alleviate inflammation, primarily through the inhibition of neutrophils (Maslowski et al., 2009; Smith et al., 2013; Bartoszek et al., 2020). Given our observation of chemokine-driven neutrophil inflammation in non-responders, downregulation of FFAR2 may further disinhibit neutrophil activation, contributing to sustained inflammation. However, there is still a lack of research on FFAR2 concerning IBD and biological agents. Only one study on another family member, FFAR4, showed that its absence alleviates colitis via Treg regulation (Zhu et al., 2022), but direct evidence for FFAR2 in UST LOR is lacking.

This study found that CD patients with baseline upregulation of genes such as ITGA2B are more likely to lose response to UST treatment. Despite non-significant qPCR results for ITGB3, it showed a similar upward trend to transcriptomics. The functional signature of the cyan module also provides mechanistic insights into UST resistance. The upregulation of ITGA2B and ITGB3 promotes the assembly and functional activation of the platelet membrane surface integrin αIIbβ3, which is encoded by the ITGA2B and ITGB3 genes, thereby inducing platelet activation and aggregation. Upon platelet activation, pro-fibrotic factors such as transforming growth factor-beta (TGF-β), serotonin, and platelet-derived growth factor (PDGF) are released. These factors activate intestinal fibroblasts and promote the deposition of extracellular matrix components like collagen, thereby driving intestinal fibrosis (Thachil, 2015; Nicolai et al., 2024). In the KEGG enrichment analysis, we enriched the ECM-receptor interaction pathway, which serves as a crucial bridge connecting inflammatory damage and intestinal fibrosis structure. Additionally, intestinal fibrosis is often accompanied by vascular injury. The platelet activation mediated by ITGA2B/ITGB3 is a key step in vascular repair, and we also enriched the platelet activation pathway. If repair is excessive, it may lead to perivascular fibrosis, thereby exacerbating intestinal wall fibrosis. Thus, upregulation of ITGA2B and ITGB3 may be related to the occurrence of intestinal fibrosis and strictures. ITGA2B has been linked to refractory CD through neoantigen-driven autoimmune responses (Zhai et al., 2023). However, none of those studies reported UST LOR.

This study is the first to report that KCNJ15 is significantly upregulated in UST LOR, with qPCR results showing significant differences. KCNJ15 has been proposed as a potential biomarker in immune-mediated diseases including CD (Wu et al., 2022; Zhang et al., 2023). Previous studies (Liu et al., 2019) have found that the inhibitory effect of KCNJ15 overexpression is regulated by its influence on the epithelial-mesenchymal transition (EMT) process and the expression of matrix metalloproteinase (MMP)-7 and p21. This pathway may also involve the excessive deposition and pathological remodeling of the extracellular matrix (ECM) in intestinal tissues, which is associated with the progression of intestinal fibrosis and stricture. Additionally, studies have shown that KCNJ15 can be expressed in immune cells, such as macrophages and T cells (Zhang et al., 2023). While our results demonstrate differential KCNJ15 expression between groups and its enrichment in the ECM-receptor interaction pathway, no definitive literature establishes a direct correlation between KCNJ15 and intestinal fibrosis or UST LOR. Thus, further investigation is warranted to elucidate the underlying complex mechanisms.

Chronic inflammation can lead to persistent damage to intestinal tissue and dysregulation of repair, resulting in excessive deposition of extracellular matrix and the formation of fibrosis, ultimately causing intestinal strictures and obstructions. In our retrospective clinical analysis, intestinal strictures were identified as an independent risk factor for UST LOR. In the trait-module association analysis, the cyan module, the one most strongly associated with UST LOR, was also most highly correlated with intestinal strictures. Functional enrichment of this module highlighted pathways related to platelet activation, ECM-receptor interaction, and NET formation, aligning with our KEGG findings (ECM-receptor interaction, focal adhesion, IL-17 signaling) and GO terms (cell chemotaxis, chemokine binding). These data suggest a coordinated inflammatory-fibrotic transcriptional program. At baseline, non-responders exhibited downregulation of FFAR2 alongside upregulation of ITGA2B, SOCS3, and KCNJ15; upregulation of ITGA2B and KCNJ15 may promote fibroblast activation and ECM deposition, suggesting the possibility of a “pro-fibrotic immune microenvironment” associated with strictures. Multiple clinical studies have consistently demonstrated that intestinal strictures predict poor UST response, including a pediatric real-world study (Mitchel et al., 2025), *post hoc* analysis of three large CD trials (Narula et al., 2021), several independent cohorts (Tang and Gao, 2022; Wang et al., 2024), and quantitative imaging predictors of primary non-response (Bao et al., 2024; Shen et al., 2025). Consistent with our findings, Xu et al. reported that lower baseline expression of fibrotic hub genes (MUC1, LCN2, PDZK1IP1) is associated with favorable UST response, further reinforcing the link between baseline fibrotic burden and suboptimal therapeutic outcomes (Xu et al., 2024).

Only a very limited number of studies have focused on the genetic research of CD patients using UST (Aguiar Zdovc et al., 2021; He et al., 2021; Nishioka et al., 2021; Hrdlička et al., 2021; Wang et al., 2025b). The novel aspects of our study are as follows. First, unlike most bioinformatics studies based on GEO database mining, we performed RNA-seq on fresh peripheral blood samples from a real-world cohort of CD patients without mixing in UC cases. Second, we chose peripheral blood instead of commonly used intestinal or fecal samples, offering a minimally invasive and more feasible approach. Third, we are the first to report that differential expression of FFAR2, ITGA2B, SOCS3, and KCNJ15 in PBMCs is associated with UST LOR in patients with CD. These candidate genes may serve as predictive biomarkers, and some of them, along with related pathways, appear to be linked to intestinal fibrosis and strictures, warranting further validation.

The limitation of this study is the limited availability of fresh blood samples from patients receiving their first UST treatment. Given the modest sample size and the use of a relatively lenient threshold to capture potential biological signals, we acknowledge that our findings are only preliminary and require further validation with larger sample sizes. Specifically, a larger cohort is needed to perform ROC curve analysis, and the potential roles of pathways beyond the direct target of UST, such as IL-10 signaling, chemokine-driven networks, and ECM-receptor interaction, require further validation in independent cohorts. Moreover, the lack of in-depth mechanistic validation restricts a deeper understanding of the dynamic mechanisms underlying UST LOR. Given this is an initial exploratory study, future work will include functional experiments and specifically the correlation between these candidate genes and intestinal stricture phenotypes using standardized imaging.

In summary, the pathogenesis of CD involves multiple signaling pathways. Our preliminary findings indicate that the differential expression of FFAR2, ITGA2B, SOCS3, and KCNJ15 may contribute to UST LOR in patients with CD. This further substantiates the need for additional exploration of the significant functions of these genes. Further studies are necessary to validate their mechanistic roles. This research not only provides preliminary evidence to candidate genes associated with UST LOR but also suggests that these genes may influence treatment efficacy by regulating processes related to intestinal stricture and fibrosis. Currently, direct evidence is lacking, and relevant literature is limited. Future validation through cellular experiments or animal models will be essential.

## 5 Conclusion

This study explored the factors influencing UST LOR in patients with CD, ranging from clinical feature analysis to transcriptomic analysis and preliminary validation. Ultimately, we reported that the genes FFAR2, ITGA2B, SOCS3, and KCNJ15 exhibited differential expression at baseline in UST non-responders. These differentially expressed genes may serve as potential biomarkers for UST LOR. In conjunction with the results from the clinical retrospective cohort study and relevant pathway analyses, we hypothesize that UST LOR may be associated with pro-inflammatory pathways independent of UST’s action pathways, as well as with a “pro-fibrotic immune microenvironment” related to intestinal strictures. This finding requires further validation in larger-scale studies.

## Data Availability

The original contributions presented in the study are publicly available. The raw sequence data reported in this paper have been deposited in the Genome Sequence Archive (Genomics, Proteomics & Bioinformatics 2025) in National Genomics Data Center (Nucleic Acids Res 2025), China National Center for Bioinformation/Beijing Institute of Genomics, Chinese Academy of Sciences (GSA-Human: HRA014832) that are publicly accessible at https://ngdc.cncb.ac.cn/gsa-human, the accession number is HRA014832.
