# Supplementary figures and images for: Exploratory transcriptomic analysis suggests candidate genes associated with loss of response to ustekinumab in Crohn’s disease
Source: Front Genet. 2026 May 22;17:1812181. doi: 10.3389/fgene.2026.1812181 (PMC13236002; doi:10.3389/fgene.2026.1812181)

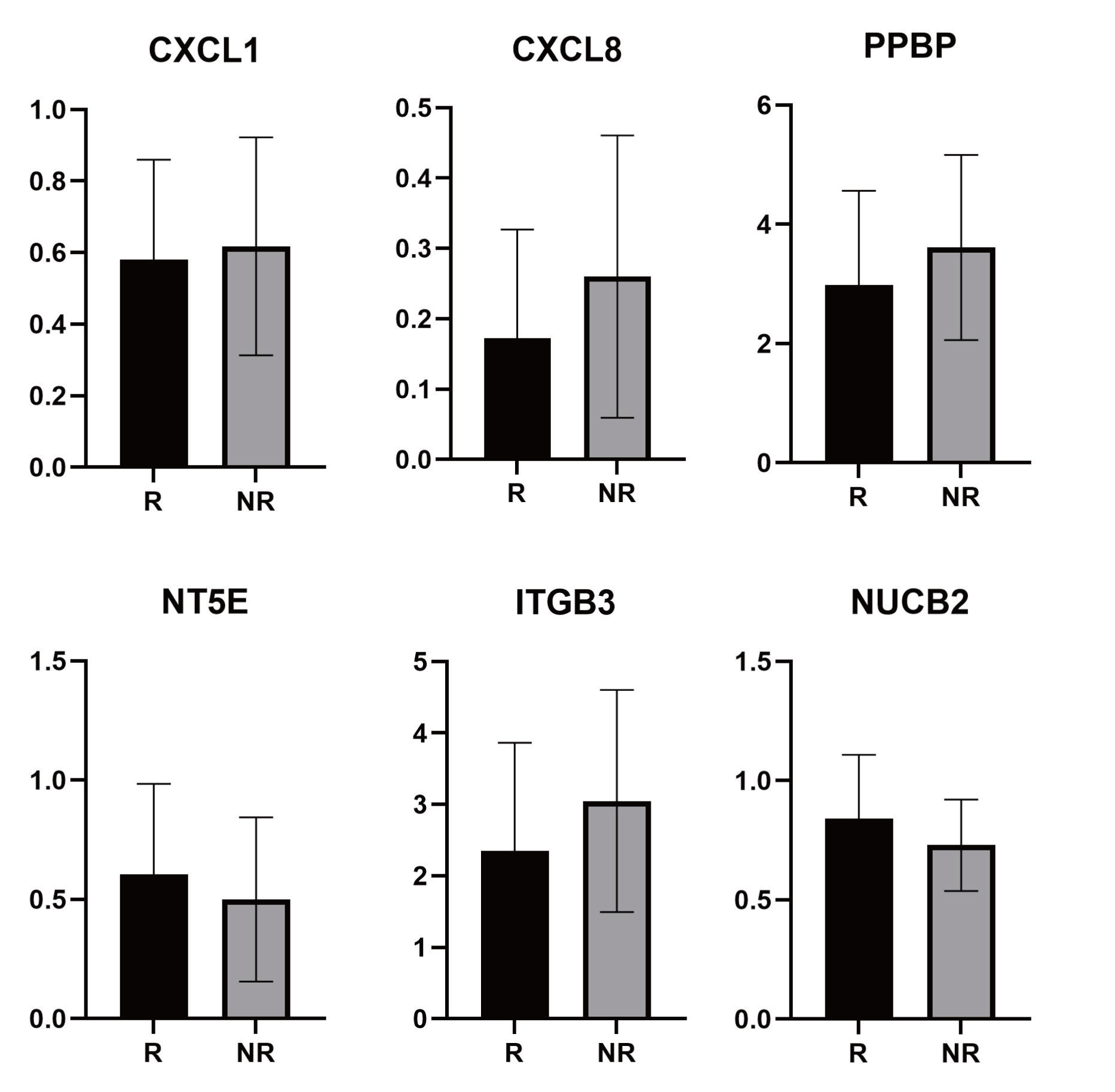

Supplement: Supplementary file 1 [file Image1.tif]
